# Ultrastructural Changes in Mitochondria in Patients with Dilated Cardiomyopathy and Parvovirus B19 Detected in Heart Tissue without Myocarditis

**DOI:** 10.3390/jpm12020177

**Published:** 2022-01-28

**Authors:** Agnieszka Pawlak, Magdalena Gewartowska, Maciej Przybylski, Mateusz Kuffner, Diana Wiligórska, Robert Gil, Zbigniew Król, Malgorzata Frontczak-Baniewicz

**Affiliations:** 1 Department of Invasive Cardiology, Central Clinical Hospital of the Ministry of Interior and Administration in Warsaw, 02-507 Warsaw, Poland; diana998@gmail.com (D.W.); scorpirg@gmail.com (R.G.); 2 Department of Applied Physiology, Mossakowski Medical Research Institute, Polish Academy of Sciences, 02-106 Warsaw, Poland; 3 Electron Microscopy Research Unit, Mossakowski Medical Research Institute, Polish Academy of Sciences, 02-106 Warsaw, Poland; mateusz.kuffner@gmail.com; 4 Department of Medical Microbiology, Medical University of Warsaw, 02-004 Warsaw, Poland; maciej@przybylscy.pl; 5 Division of Cardiology, Health Care Facility of the Ministry of Interior and Administration in Rzeszow, 35-111 Rzeszow, Poland; 6 Central Clinical Hospital of the Ministry of the Interior and Administration in Warsaw, 02-507 Warsaw, Poland; zbigniew.krol@cskmswia.pl

**Keywords:** viral infection, electron microscopy, dilated cardiomyopathy, parvovirus B19, mitochondria

## Abstract

Understanding the meaning of parvovirus B19 (PB19V) in an etiology of dilated cardiomyopathy (DCM) is difficult. Viruses change the dynamics of the mitochondria by interfering with the mitochondrial process/function, causing the alteration of mitochondrial morphology. In this study, the ultrastructural changes in the mitochondria in endomyocardial biopsy (EMB) samples from patients with DCM and PB19V were determined. Methods: The PB19V evaluation was performed in EMB specimens by real-time PCR in 20 patients (age: 28 ± 6 years). The biopsy specimens were examined by histo- and immunohistochemistry to detect the inflammatory response. The ultrastructural features of the mitochondria were evaluated by electron microscopy. Results: The presence of PB19V in the heart tissue without the presence of inflammatory process, defined according to Dallas and immunohistochemical criteria, was associated with ultrastructural changes in the mitochondria. Distinctive ultrastructural pathologies were indicated, such as the presence of mitochondria in the vicinity of the expanded sarcoplasmic reticulum with amorphous material, blurred structure of mitochondria, interrupted outer mitochondrial membrane and mitophagy. Conclusions: Extending diagnostics with ultrastructural analysis of biopsy samples provides new knowledge of the changes associated with the presence of PB19V in the heart tissue. The observed changes can be a basis for searching for the damage mechanisms, as well as for new therapeutic solutions.

## 1. Introduction

In 50% of cases, dilated cardiomyopathy (DCM) is associated with the presence of a virus in the heart tissue [1,2]. In Europe, parvovirus B19 (B19V) is most commonly detected in the heart tissue [3,4]. PB19V is more often found in the myocardium without the presence of inflammation than in patients with diagnosed myocarditis. During the replication of several viruses, mitochondria are clustered around their replication sites, resulting in the decrease of the cellular supply routes for energy and metabolites and resulting in increased production of viral progeny. In a viral infection, viruses generate cellular stress, which causes mitochondrial redistribution. PB19V, as fast-replicating viruses, easily cope with cellular metabolic dysfunction [5]. The influence of fast-replicating viruses on the mitochondria results in modifications of the morphology, number, and localization of these organelles. The mitochondria regulate cell metabolism, bioenergetics, cell death, innate immune signaling, and cellular homeostasis through processes such as fusion or fission [6,7]. The morphology of the mitochondria can also be modified by the uncontrolled release of reactive oxygen species (ROS), which cause changes in the structure of cellular lipids and proteins by promoting the damage of the mitochondrial membranes’ structure [8,9]. Another source of mitochondrial quality control is the selective degradation of dysfunctional mitochondria through their autophagy (mitophagy) [10]. The course and intensity of these processes depend on the level of the metabolic needs of the cell, which is vastly increased during viral infection [11].

Currently, the role of the mitochondria in the pathology of DCM has aroused the interest of many researchers [12]. The ultrastructural assessment shows the changes in the heart tissue of patients with DCM and PB19V infection, including changes in the morphology, number, and localization of mitochondria.

The ultrastructural changes in the mitochondria have not yet been described in patients with PB19V presence in the heart tissue. Publications on experimental studies conducted on animals or cell cultures involve only animal parvoviruses. As there is no animal model for PB19V infection, and in vitro propagation of the virus requires human primary erythroid cell culture, there is only limited data available concerning more detailed analysis of the PB19V infection at cellular or subcellular level.

The ultrastructural analysis of biopsy samples, utilizing electron microscopy, has not yet been used yet for the analysis of the changes associated with B19V presence in the heart tissue. The observed abnormalities can be the basis for searching the mechanisms responsible for heart tissue damage in DCM associated with PB19V, as well as new therapeutic solutions.

The aim of the study was to analyze ultrastructural changes in mitochondria in patients with DCM and the presence of PB19V DNA in the heart tissue.

## 2. Materials and Methods

### 2.1. Study Population

The study population consisted of 27 patients with clinically suspected myocarditis or inflammatory DCM, admitted to the Department of Invasive Cardiology of the Central Clinical Hospital of the Ministry of Interior and Administration in 2015–2017.

The study population included three groups: 10 patients with DCM and the confirmed presence of PB19V DNA in the myocardial tissue, 10 patients with DCM and without PB19V, and a control group consisting of 7 patients, who were eligible for suspected myocarditis but EMB did not confirm either tissue inflammation or PB19V in the heart tissue.

Before EMB, all patients underwent physical examination, and the following tests were performed: laboratory studies (C-reactive protein, leukocytes, N-terminal-pro-brain natriuretic peptide (NT-pro-BNP), and troponin I (TnI)), 12-lead electrocardiogram (ECG), echocardiography for the measurement of left ventricular end-diastolic diameter (LVEDD), and ejection fraction (LVEF) with planimetric Simpson’s method, intraventricular septum diameter, and posterior wall diameter. Coronary angiography was performed to exclude relevant coronary artery disease (diameter stenosis > 50%).

The study has been approved by the local ethics committee of the Central Clinical Hospital of the Ministry of Interior, Warsaw, Poland. The study protocol conformed to the ethical guidelines of the Declaration of Helsinki and its later amendments. Informed written consent was obtained from every study participant prior to their inclusion in the study.

### 2.2. Endomyocardial Biopsy

Endomyocardial biopsy (EMB) was performed via femoral approach, using a 7-Fr catheter bioptome (Cordis, Johnson & Johnson, New Brunswick, NJ, USA), under control of an X-ray scope and during continuous monitoring of ECG. Six specimens were collected from the left ventricle. All patients underwent control echocardiography examination after EMB.

Three paraffin-embedded specimens were stained with hematoxylin & eosin and with Masson’s trichrome. The categories of acute and borderline MCI according to the Dallas criteria were assessed jointly [13]. Localization and identification of mononuclear cell infiltrates were performed with antibodies against leukocyte common antigen (M0701), CD3 (M7254), CD4 (M7310), CD8 (M7103) for T cells, and CD68 (M0876) for macrophages (DAKO, Germany). Based on immuno-histological criteria, inflammation was considered when focal or diffuse mononuclear infiltrates (>14 leukocytes/mm^2^, CD3+ T lymphocytes > 7/mm^2^ and CD68+ macrophages < 4/mm^2^) were detected in the myocardial cells [14]. Two EMB specimens were snap-frozen in liquid nitrogen for the molecular pathological exam, including the real-time PCR method for PB19V detection. One EMB specimen was dedicated for assessment via electron microscopy.

### 2.3. Quantitative Real-Time PCR (qPCR)

DNA was isolated from two frozen myocardial biopsy samples (20–50 µg of quick-frozen tissue) with the NucleoSpin Tissue DNA kit (Macherey-Nagel, Dueren, Germany). Primers and a dual-labeled probe, specific for VP1 gene of the parvovirus B19, were used for amplification. All PCR reactions were performed with primers and probe specific for human glucose-6-phosphate dehydrogenase for the confirmation of the DNA isolation from the tissue and for the normalization of viral DNA copy number. A detailed description of the method used for B19V detection and quantitation has been published previously [15].

### 2.4. Transmission Electron Microscopy (TEM)

The biopsy material was sequentially fixed in 2% paraformaldehyde, 2.5% glutaraldehyde in cacodylate buffer, and 1% osmium tetra-oxide with potassium ferricyanide, for 2 h. Then, the samples were dehydrated in a series of ethanol and embedded in resin. The ultra-thin sections were stained with uranyl acetate and lead citrate. Images were acquired using JEM-1011 EX (Jeol, Tokyo, Japan) transmission electron microscope equipped with MORADA camera and iTEM 1233 software.

Semiquantitative analysis was performed using 10 randomly selected photographs of the heart specimens from each patient’s biopsy. The following morphological features were evaluated: blurred structure of mitochondria, partial loss of mitochondrial cristae, autophagy of mitochondria, increased electron density of mitochondrial matrix, and disrupted outer mitochondrial membrane. The intensity of the features was assessed in a 4-point scale (0—feature is absent, 1—low intensity, 2—moderate, 3—high intensity of a feature). Electron microscopy data are shown as median, with interquartile range (IQR) from 10 fields of view from each patient and of the results obtained from all patients in each group.

### 2.5. Statistical Methods

Continuous variables were expressed as means ± SD or as their logarithmic values, continuous variables with a skewed distribution as a median with lower and upper quartiles, and categorical variables as a percentage. Intergroup differences were analyzed with a Student’s *t*-test, whereas ANOVA was used for multiple group comparisons for the populations with normal distribution; otherwise ANOVA on ranks was used. The Shapiro-Wilk test was used to check whether the samples represented a normal distribution. Frequencies were compared using the χ^2^ test. All tests were 2-sided with a significance level of *p* ≤ 0.05. A commercial statistical package (SPSS v.13.0; IBM, Armonk, NY, USA) was used for all statistical analyses.

## 3. Results

### 3.1. Study Population

The characteristics of the study population are presented in Table 1. All patients included in the study were young males without any comorbidities such as hypertension, kidney disease, hyperlipidemia, or diabetes. Patients with DCM and PB19V in the heart tissue had significantly worse echocardiographic parameters (LVEF and LVEDD) in comparison to the control group, but comparable to a group of with DCM and without PB19V (LVEF 21.7 ± 6.1 vs. 61.3 ± 2.3 and 23.3 ± 10.4%; LVEDD 62.3 ± 9.5 vs. 47.7 ± 2.1 vs. 64.0 ± 7.2 mm, respectively) (Table 1). Patients with PB19V were characterized by the highest NT-pro-BNP and CRP levels. None of the patients, irrespective of the group, met the histopathological criteria of myocarditis. Histological H/E and Masson’s trichrome staining for fibrosis as well as immunohistochemical labeling for immunocompetent cells are available as a supplementary file (Figure S1). Time from the last episode of antecedent flu-like illness to EMB collection was shorter in the control group (3 ± 3 months) than in the group with DCM and with or without PB19V (6 ± 3 months).

### 3.2. Electron Microscopy Studies

#### 3.2.1. Group I—LVEF ≥ 50%, without PB19V DNA

**Figure 1:**
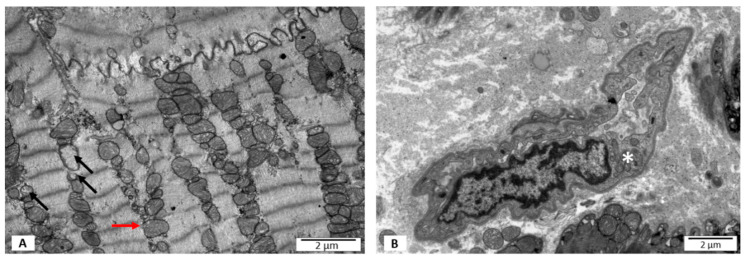
Electron microscopy images. Ultrastructural features of heart biopsies from patients with EF ≥ 50% and no virus detected. (**A**) Loss of mitochondrial cristae marked with black arrows; disrupted outer mitochondrial membrane marked with red arrow. (**B**) No features of necrosis; hypertrophic endothelium marked with an asterisk.

The ultrastructure assessment showed the presence of a few mitochondria characterized by the blurred structure of the mitochondrial membrane. Similarly, mitochondria with disrupted outer mitochondrial membrane were observed rarely. Occasionally, partial loss of mitochondrial cristae was visible (Figure 1A). The size, shape, and distribution of the mitochondria were different from normal cardiomyocytes only in a few cases (Table 2). The endothelium of local capillary vessels in the extracellular space presented hypertrophy features (Figure 1B).

#### 3.2.2. Group II—LVEF < 50%, without PB19V DNA

**Figure 2:**
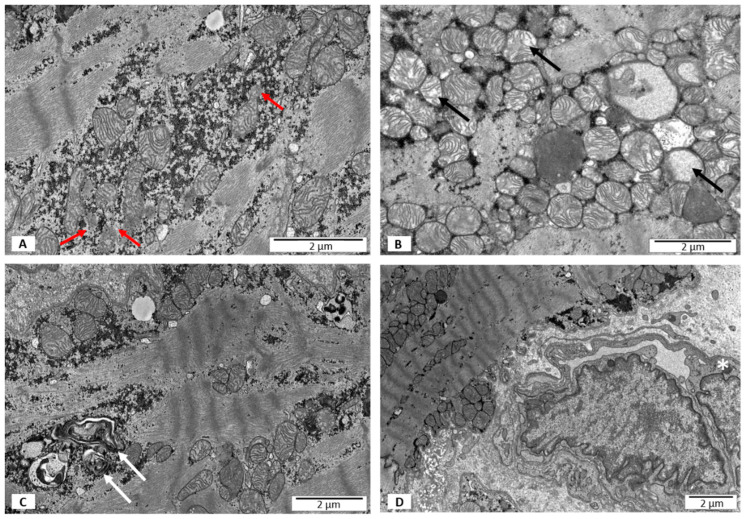
Electron microscopy images. Ultrastructural features of heart biopsies from patients with EF < 50% and no virus detected. (**A**) A disrupted outer mitochondrial membrane marked with red arrows; (**B**) partial loss of mitochondrial cristae and swollen mitochondria marked with black arrows; (**C**) lysosomes and autophagous figures containing remnants of mitochondrial cristae marked with white arrows; (**D**) no features of necrosis; hypertrophic endothelium marked with an asterisk.

In this group of patients, a small number of the mitochondria in cardiomyocytes had blurred mitochondrial membrane, as in group I. Disruption of the outer mitochondrial membrane was more often visible when compared to group I (Figure 2A). Additionally, there was an increased number of mitochondria with partial loss of cristae (Figure 2B). Some of the mitochondria were swollen (Figure 2B). Lysosomes and autophagosomes containing remnants of mitochondrial cristae were present in the cardiomyocyte cytoplasm in the proximity of the nucleus (Figure 2C). A significant number of the mitochondria demonstrated pleomorphism (Figure 2A), and some of the mitochondria closely adhered to each other (Figure 2B). More often than in group I, mitochondria were localized in the perinuclear area (Table 2). The local capillary vessels had a normal morphological structure (Figure 2D).

#### 3.2.3. Group III—LVEF < 50%, with B19V DNA

**Figure 3:**
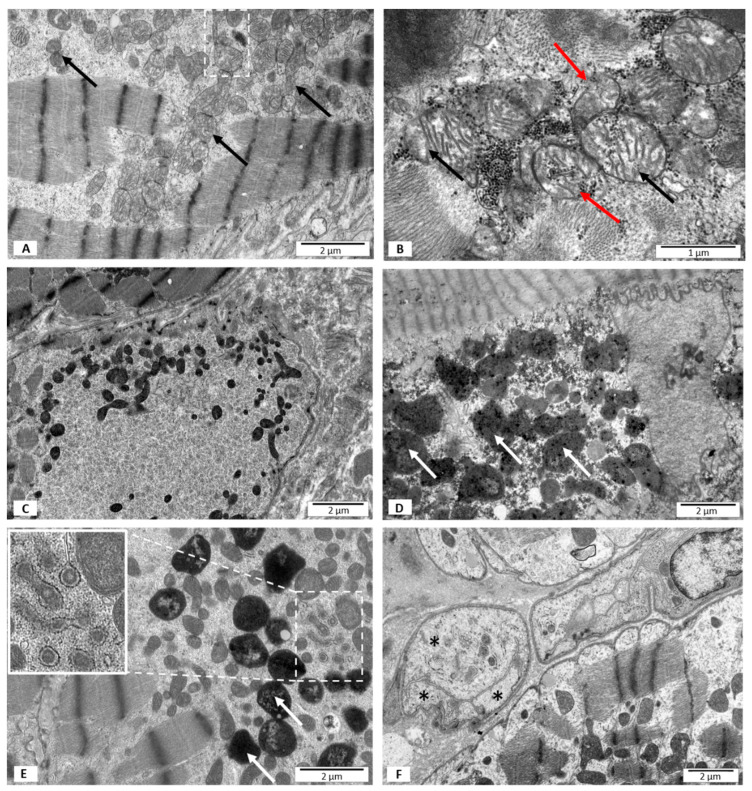
Electron microscopy images. Ultrastructural features of heart biopsies from patients with EF < 50% and PB19 virus detected. (**A**) A blurred structure of mitochondrial membranes marked with black arrows; mitochondria showing morphological features of fission marked with frame; (**B**) a disrupted outer mitochondrial membrane marked with red arrows; glycogen granules in the mitochondrial matrix are visible. Mitochondria showing morphological signs of swelling marked with black arrows; (**C**) increased electron density of the mitochondria; (**D**,**E**) high mitophagy rate; mitophagosomes marked with white arrows; (**E**) sarcoplasmic reticulum with the amorphous material marked with frame and shown in the insert; (**F**) blood vessel with features of necrosis marked with asterisks.

In the material collected from this group of patients, many disorders of the mitochondrial ultrastructure were observed. The blurred structure of mitochondrial membranes was the most characteristic and common feature (Figure 3A,B). In comparison to group I and II, there was a noticeably higher number of mitochondria with disrupted outer mitochondrial membrane (Figure 3B). The matrix of some mitochondria contained glycogen granules (Figure 3B). Moreover, some mitochondria showed morphological signs of swelling (Figure 3B). The partial loss of mitochondrial cristae was observed with the same frequency as in group I, and less often than in group II. In this group, in contrast to group I and II, there was a number of mitochondria presenting a homogenous matrix with increased electron density (Figure 3C). The second characteristic feature of the cardiomyocytes in this group was the presence of numerous mitophagosomes and phagolysosomes in the perinuclear area (Figure 3D,E) which was in contrast to group I, where no morphological features of mitophagy were observed (Table 2, Figure 3A). In group III, the most diverse sizes and shapes of the mitochondria were observed, and some mitochondria showed features of fission. Similarly to group II, there were a number of closely adherent mitochondria (Figure 3A). Mitochondria in the vicinity of the expanded sarcoplasmic reticulum, within which the amorphous material was visible, were found only in group III (Figure 3E). The majority of local capillary vessels in the extracellular space had normal ultrastructural structure; however, some showed morphological features of necrosis (Figure 3F).

## 4. Discussion

Dilated cardiomyopathy (DCM) is a significant diagnostic and therapeutic challenge because of unfavorable prognosis, frequent hospitalizations, and negative social and economic aspects [16]. Factors which lead to the development of DCM include cardiotropic viruses such as human herpesvirus 6 (HHV-6), human enterovirus (HEV), cytomegalovirus (CMV) and Epstein-Barr virus (HHV-4) [3]. Presence of these viruses in the heart tissue has been associated with the patho-mechanism of both myocarditis and DCM and different changes at the cellular level, which can result in various clinical consequences, such as heart failure or life-threatening arrhythmias. In the European population, the most common virus found in the myocardial tissue is parvovirus B19 (PB19V); however, the exact role of this virus in the pathogenesis of DCM is still being discussed [17]. The involvement of PB19V in the etiology of DCM, is proposed on the basis of an increased percentage of DCM patients with viral DNA in myocardial tissue when compared to a control group, despite the lack of local inflammation and almost complete absence of corresponding PB19V viremia. On the other hand, a higher level of viremia, suggesting active replication of the virus, is very rarely associated with clinical features of DCM. The virus’s ability to cause endothelial dysfunction, which triggers processes leading to the DCM development, is believed to be the main cause of the pathogenesis [18]. There are studies showing that mitochondrial damage is an important pathophysiological factor leading to myocardial dysfunction and thus to the development of heart failure [19]. Moreover, viruses are able to interact with the mitochondria and can modulate mitochondrial functions for their own benefits [20]. However, available studies do not explain the role of mitochondrial damage in the development of pathological alterations in the structure of cardiomyocyte, and thus the macroscopic consequence of myocardial dysfunction in assumed PB19V-associated DCM. The impact of PB19V on the morphology and function of the mitochondria is still little known.

To the best of our knowledge, this is the first study, which evaluates the ultrastructural changes in the mitochondria in the population of young patients with DCM accompanied by the presence of PB19V in myocardial tissue without a defined inflammatory process.

On the basis of the results of the present study, the ultrastructural changes of the mitochondria associated with the presence of PB19V are mainly blurred structure of outer mitochondrial membrane, disrupted outer mitochondrial membrane, mitophagy, and the presence of mitochondria in the vicinity of the expanded sarcoplasmic reticulum with the amorphous material.

The blurred structure of the mitochondrial membranes is the most common symptom of abnormal mitochondrial morphology in patients with DCM and PB19V. It seems that the oxidative stress generated by a viral infection, which causes an increased production of free oxygen radicals (ROS), is most likely to be responsible for this abnormality [21]. ROS cause peroxidation of membranes’ lipids and protein damage, which at the level of membrane morphology may present as the formation of a blurred structure. Another ultrastructural abnormality observed in the population of DCM and PB19V patients is the continuity disruption of the mitochondrial membranes. This effect is confirmed by the electron microscopy images of the mitochondria, which show the presence of glycogen granules within the mitochondrial matrix. The presence of swollen mitochondria also indicates an abnormal structure of mitochondrial membranes. The blurred structure of the mitochondrial membranes is often accompanied by a disrupted outer membrane. This feature is more frequent in group II than in group I, despite the fact that a similar frequency of the blurred membrane structure is observed in these groups. It should be noted that group II includes patients with advanced heart failure in echocardiographic imaging (LVEF 23%, LVEDD 64 mm), which indirectly suggests the possibility of energy deficiencies. Accompanying the echocardiographic image, the increased number of small mitochondria and morphologically altered mitochondria in ultrastructural studies confirm these assumptions. The numerous small mitochondria observed may be the result of fission processes. Increased permeability transition, ROS production, mitophagy, cell death, and decreased oxidative capacity, defining fission processes, which can be strongly expressed in insufficient heart, can contribute to the loss of membrane continuity [22].

The increased electron density of the mitochondrial matrix in the electron microscopic images is also associated with the increased production of ROS [23]. In this study, increased electron density was observed in group III. This image is consistent with a significantly more often observed blurred structure of the mitochondrial membranes, also present in this group. These images represent the morphological expression of the increased ROS production generated by the virus presence.

The analyzed biopsy material showed increased loss of mitochondrial cristae significantly more often in the group of patients with DCM, but without the virus presence in the heart tissue. This pathology was observed with equal frequency in patients with preserved LVEF and in patients with DCM and PB19V. Infrequent occurrence of mitochondrial cristae damage in the ultrastructural analysis in group III may indicate a protective role of the virus in relation to the structures responsible for cells’ energy generation. It is believed that one of the primary tasks of PB19V is to maintain cellular energy homeostasis. This result requires further analysis.

A mitophagy is the main cellular process responsible for controlling mitochondria quality. During this process, the damaged mitochondria are directed to lysosomes for degradation. In this study, mitophagy is the second characteristic feature after the blurred structure of mitochondrial membranes in the group of patients with DCM and PB19V. The obtained images confirm the data on the promotion of mitophagy by PB19V reported in the literature [24]. Growing evidence suggests that viruses use mitophagy to promote survival, using various strategies to manipulate this process, to eliminate critical outer-membrane mitochondrial immune regulatory proteins in order to attenuate the host’s immune response [25,26]. This correlates with the studies’ results, which have shown that autophagy can modulate the course of immune and adaptive reactions by regulating the differentiation of immunologically competent cells, their survival, phagocytosis, antigen presentation, degranulation or cytokine production, which in the case of PB19V infection leads to the promotion of viral infection [27,28,29]. In the present study, the morphological features of mitophagy in cardiomyocytes, which was not accompanied by infiltration of inflammatory cells in the tissue, were observed. The absence of the inflammatory process in the biopsy specimens may be the result of the increased autophagy. No morphological features of apoptosis have been observed, although apoptosis is often reported in viral infections. It is possible that the observed image is due to the early stage of viral infection, in which viruses prevent apoptosis in order to avoid the host immune response and promote cell replication and autophagy of the mitochondria. Patients analyzed in this study had a short medical history (several months), which may partially explain the lack of morphological features of cardiomyocytes’ apoptosis. The physiological and pathological significance of mitophagy in the heart is unclear. On the one hand, in the pathogenesis of heart disease, during this process damaged mitochondria can be removed, which has a positive effect on the heart function; on the other hand, excessive mitophagy leads to heart damage. However, it seems that the cardiomyocytes’ remodeling at the cellular level, resulting from energy metabolism changes, causes stress in the entire cardiomyocyte and changes in the structure of the contractile apparatus and the cytoskeleton, leading to the development of DCM.

A phenomenon observed only in group III is the presence mainly of small mitochondria around the endo-plasmatic reticulum (ER) with the amorphous material in the lumen. A diversity of external stimuli, including pathogen viruses’ invasion, have been shown to impose stress on the ER by leading to alterations such as failure of post-translational modifications and a general increase in protein synthesis [30,31]. Perturbations of ER homeostasis cause an accumulation of misfolded and unfolded proteins in the ER’s lumen, activating complex signaling pathways that deal with the misfolded and unfolded proteins, referred to as the unfolded protein response [32]. Viruses are also known to alter host cell Ca^2+^ signaling and metabolic pathways to accelerate their replication. Several viruses induce intracellular Ca^2+^ concentrations to increase uptake by cellular Ca^2+^ stores such as the ER. The proper functioning of the ER and mitochondria depends on the concentration of Ca^2+^ ions, which is an essential component of the virus–host interaction. The large gradient across membranes allows viruses to modulate this buffered environment easily in order to induce productive infection and evade host immune response [33].

Previous studies have shown that in persistent myocardial infection, PB19V is present in capillary endothelial cells [17,32]. The authors speculate that, due to the high viral load in the heart, PB19V infection of the endothelial cells was sufficient to induce impaired coronary microcirculation, while the result is ambiguous in the ultrastructural assessment. In group III, in the extracellular space, most often capillary vessels had normal ultrastructure, and only a few showed morphological features of necrosis. Perhaps the lack of capillary vessel changes results from the lack of an inflammatory reaction in the heart tissue in patients included in the study.

## 5. Conclusions

The presence of PB19V in the myocardial biopsy, without histological and immunohistochemical features of myocarditis in patients with DCM, is associated with ultrastructural changes of the mitochondria, which are mainly blurred mitochondrial structure, broken outer mitochondrial membrane continuity, mitophagy, and the presence of mitochondria in the vicinity of the expanded sarcoplasmic reticulum with the amorphous material. The observed changes can be the basis for studies related to damage mechanisms and new therapeutic options. Therefore, further studies are needed to identify the causes of cardiomyopathy development and to identify changes at the subcellular level, as well as to relate these to changes observed at the clinical level.

## 6. Study Limitations

An important limitation of the study was the small population of the study, which consisted of 27 patients. Moreover, the studied population was highly selective (consisted of males, average age 27–28 years, patients without any comorbidities) in order to exclude the influence of many variables on the changes in the ultrastructure of mitochondria. This can be a limitation as well as an advantage. A very limited population enables exclusion of the influence of many factors, such as age, comorbidities, and sex, on the ultrastructural changes in cardiomyocytes and to evaluate only the influence of PB19V in the heart tissue. Additionally, endomyocardial biopsy (six specimens from each patient) and electron microscopy analysis is an assessment of only a few small sections of the heart tissue. More studies on larger populations are needed to confirm and broaden the results of this study, which can have impact on patients’ prognosis and can be a basis for searching for damage mechanisms as well as new therapeutic solutions.

## Figures and Tables

**Table 1:** Study population.

Parameters	Control Group (*n* = 7)	Patients with DCM without Virus (10)	Patients with DCM and Virus (10)	*p*
Male, *n* (%)	7 (100)	10 (100)	10 (100)	0.97
Age, years	28.0 ± 3.2	27.0 ± 6.3	28.7 ± 6.5	0.79
NYHA Class, *n* (%)				
Class I	2 (29)	1(10)	1 (10)	0.14
Class II	5 (71)	7 (70)	6 (60)	0.76
Class III	0	2 (20)	3 (30)	0.07
Class IV	0	0	0	0.98
LVEF, (%)	61.3 ± 2.3	23.3 ± 10.4	21.7 ± 6.1	0.01 *
LVEDD, (mm)	47.7 ± 2.1	64.0 ± 7.2	62.3 ± 9.5	0.01 *
IVSDD, (mm)	10.5 ± 0.7	9.3 ± 1.1	10.3 ± 1.1	0.08
PWDD, (mm)	10.0 ± 0.9	9.3 ± 1.1	10.0 ± 1.0	0.11
NT-pro-BNP, (pg/dL)	398.6 ± 123.2	609.3 ± 126.3	2281.6 ± 313.4	0.001 **
CRP > 5 mg/L, *n* (%)	13.9 ± 4.2	10.2 ± 3.9	33.8 ± 7.2	0.05 **
Leukocytes > 10.000/uL, *n* (%)	6.3 ± 1.4	7.4 ± 1.3	6.5 ± 1.8	0.12
TroponinI > 0.07 ng/mL, *n* (%)	19.6 ± 4.3	17.7 ± 3.5	23.3 ± 5.3	0.08
Diseases, *n* (%)				
Hypertension	0	0	0	0.98
Hyper-lipidemia	0	0	0	0.97
Diabetes	0	0	0	0.97
Renal failure (MDRD < 40 mL/min/1.73 m^2^)	0	0	0	0.97
History of smoking	1 (14)	1 (10)	2 (20)	0.32
Medication, *n* (%)				
ACE-I i/lub ARB	7 (100)	10 (100)	10 (100)	0.98
Beta-blockers	2 (29)	7 (70)	10 (100)	0.05 *
Aldosteron antagonist	0	3 (30)	7 (70)	0.08
Loop diuretics	0	7 (70)	10 (100)	0.04
Statin	0	7 (70)	10 (100)	0.04
Digoxin	0	0	33	0.07
EMB results, *n* (%)				
Acute myocarditis	0	0	0	0.98
Borderline myocarditis	0	0	0	0.98
No myocarditis	7 (100)	10 (100)	10 (100)	0.98
Detection of viral genome				
PB19 [no of copy]	0	0	6670	0.001 **

Data are presented as a number of patients (*n*) and percentage of the population (%). DCM—dilated cardiomyopathy, EMB—endomyocardial biopsy, NYHA—New York Heart Association, LVEF—left ventricular ejection fraction, LVEDD—left ventricular end-diastolic diameter, IVSDD—intraventricular septum diastolic diameter, PWDD—posterior wall diastolic diameter, NT-pro-BNP—N-terminal pro B-type natriuretic peptide, CRP—C-reactive protein, ACE-I—angiotensin-converting-enzyme inhibitor, ARB—angiotensin receptor blocker. * *p* > 0.05 between control group and groups with DCM with and without PB19V, ** *p* > 0.05 between group with DCM and PB19V vs. control group and group with DCM without PB19V.

**Table 2:** Ultrastructural changes in the mitochondria in patients with DCM and PB19V in myocardial tissue.

	Group III Patients with LVEF ≥ 50%, PB19V Negative (Control) *n* = 7	Group II Patients with DCM, LVEF < 50% PB19V Negative *n* = 10	Group III Patients with DCM, LVEF < 50% PB19V Positive *n* = 10	*p*
Blurred structure of mitochondria [median (IQR)]	1(1;1)	1 (1;1)	3 (2;3)	0.0001 *
Increased electron density of mitochondrial matrix [median (IQR)]	0 (0;0)	0 (0;0)	1 (1;1)	0.01 *
Disrupted outer mitochondrial membrane [median (IQR)]	1(1;1)	1 (1;2)	2 (1;2)	0.001 **
Loss of mitochondrial cristae [median (IQR)]	1 (1;1)	1 (1;2)	1 (1;1)	0.01 ***
Swelling of mitochondria [median (IQR)]	0 (0;0)	1 (1;1)	2 (2;3)	0.001 **
Autophagy of mitochondria [median (IQR)]	0 (0;1)	1 (1;2)	2 (2;3)	0.0001 **

* *p* < 0.05 between group III vs. control group and group II; ** *p* < 0.05 between control group vs. group II and group II vs. group III and control group vs. group III; *** *p* < 0.05 between group II vs. control group and group III. The degree of mitochondrial damage was determined according to the scale: 0—no feature; 1—prevalence of the feature: 1–25%; 2—prevalence of the feature: 26–50%; 3—prevalence of the feature: 51–100%.

## Data Availability

The data that support the findings of this study are available on request from the first author.

## Supplementary Materials

The following supporting information can be downloaded at: https://www.mdpi.com/article/10.3390/jpm12020177/s1, Figure S1: Heart tissue samples from patients with DCM and PB19V: A–infiltration of leucocytes; B–infiltration of CD3 positive cells; C–infiltration of CD68 positive cells. All immunostainings performed with Dako anti human antibodies and EnVision HRP system (DaKo, Germany), magnification ×40, D–myocytolysis in hematoxylin and eosin staining, magnification ×100; E–fibrosis in Masson’s trichrome staining, magnification ×100.
