# Supplementary material for: Ultrastructural Changes in Mitochondria in Patients with Dilated Cardiomyopathy and Parvovirus B19 Detected in Heart Tissue without Myocarditis
Source: J Pers Med. 2022 Jan 28;12(2):177. doi: 10.3390/jpm12020177 (PMC8880015; doi:10.3390/jpm12020177)
Supplement: Supplementary file 1 [file jpm-12-00177-s001.zip › jpm-1525217-supplementary.pdf]

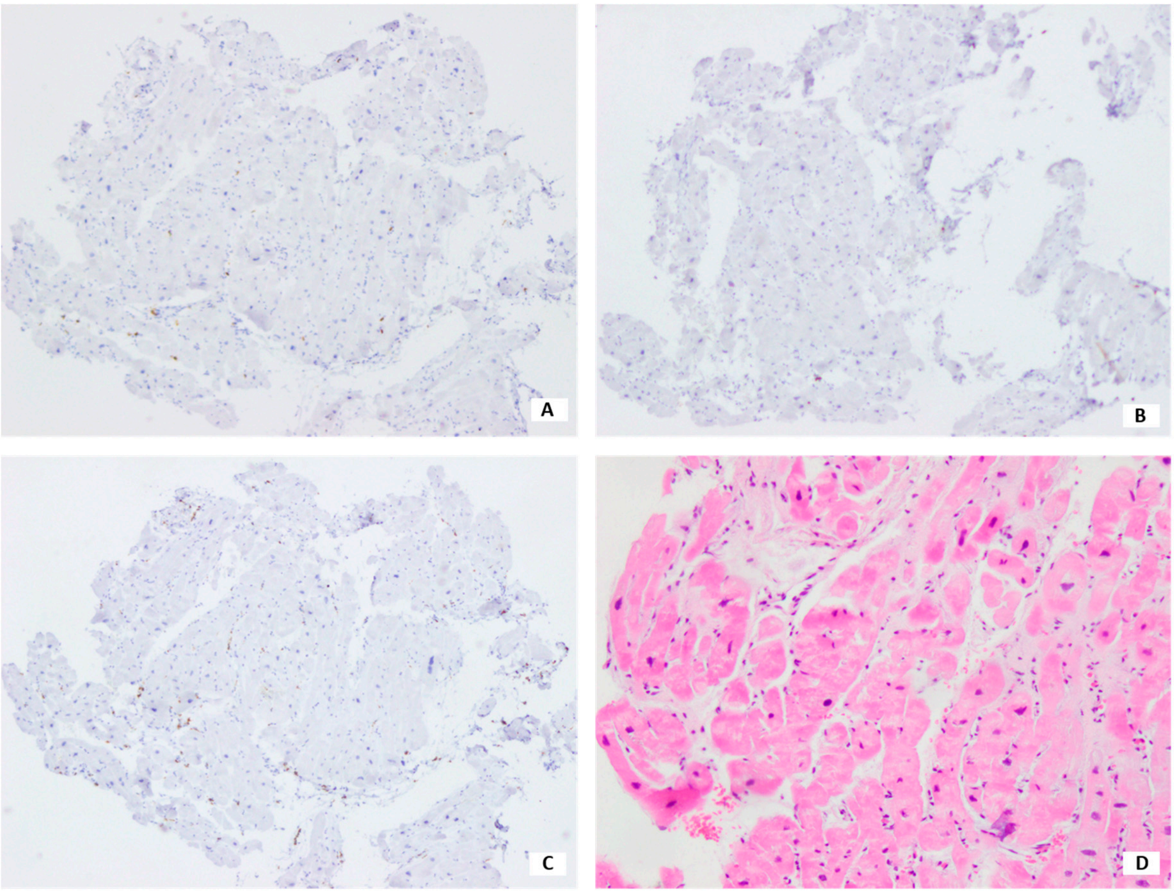

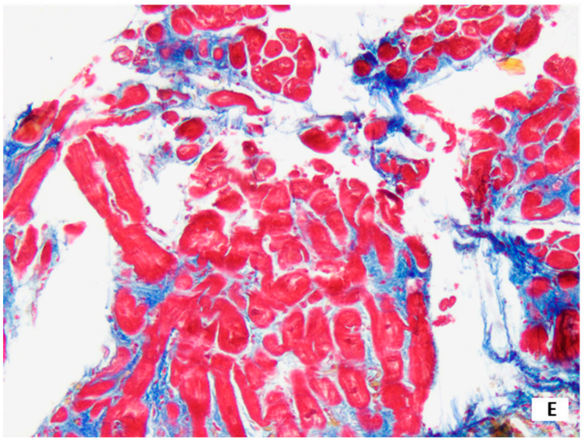

Figure S1. Heart tissue samples from patients with DCM and PB19V: A – infiltration of leucocytes; B - infiltration of CD3 positive cells; C - infiltration of CD68 positive cells. All immunostainings performed with Dako anti human antibodies and EnVision HRP system (Dako, Germany), magnification x40, D - myocytolysis in hematoxylin and eosin staining, magnification x100; E - fibrosis in Masson's trichrome staining, magnification x100.
